# Safe and sustainable by design with ML/AI: A transformative approach to advancing nanotechnology

**DOI:** 10.3762/bjnano.17.11

**Published:** 2026-01-16

**Authors:** Georgia Melagraki

**Affiliations:** 1 Hellenic Military Academy, Vari, Greece

**Keywords:** digital twins, machine learning, materials informatics, safe and sustainable by design

## Abstract

Nanotechnology is revolutionizing different sectors such as medicine, energy, defence, and environmental science by enabling the development of materials and technologies with exceptional precision and efficiency. From advanced drug delivery systems to clean energy solutions, the applications of nanotechnology are diverse and transformative. However, these innovations are accompanied by complex challenges regarding safety and sustainability for both the nanoscale materials themselves and for the products containing them. The growing complexity of engineered nanomaterials calls for proactive strategies to mitigate potential risks while maintaining their functional benefits. The "Safe and Sustainable by Design" (SSbD) concept addresses these challenges by embedding safety measures and sustainability considerations into the earliest stages of material development. Advances in machine learning (ML) and artificial intelligence (AI) have further enhanced the effectiveness of SSbD by providing predictive modelling, risk assessment, decision-making tools, and the ability to computationally screen candidate materials before producing them. This perspective article highlights how ML and AI are driving the evolution of SSbD in nanotechnology, focussing on predictive toxicology, materials informatics, lifecycle analysis, and the pivotal role of digital twins. It also explores current challenges, emerging opportunities, and the path forward for integrating ML/AI-driven SSbD frameworks into regulatory and industrial practices.

## Introduction

Nanotechnology has fundamentally changed the landscape of materials science, offering unprecedented opportunities to design and develop nanomaterials with unique, tailored properties. These advances have significantly impacted diverse industrial sectors, including healthcare, energy, environmental remediation, and defence. For instance, nanoparticle-based drug delivery systems have enabled targeted therapies for cancer, minimizing side effects while enhancing therapeutic efficacy [1–2]. In the energy sector, nanostructured materials have enhanced the performance and energy density of batteries and solar cells, providing more sustainable and efficient solutions [3]. Additionally, engineered nanomaterials (ENMs) have been employed for environmental applications, such as water purification and pollutant removal, addressing some of the most pressing ecological challenges [4–5]. Nanotechnology has significant applications in defence [6], particularly in the development of lightweight, high-strength materials for advanced armour systems and protective gear. For example, nanostructured ceramics and nanocomposites enhance ballistic protection while reducing weight, improving mobility for soldiers [7]. Additionally, nanosensors can detect chemical and biological threats in real time, providing critical situational awareness on the battlefield [8]. These innovations improve operational capabilities and safety in defence environments.

However, the rapid development of ENMs and their wide-scale application across sectors has introduced significant concerns regarding their environmental, health, and safety (EHS) risks. The unique physicochemical properties of ENMs, including their high surface-to-volume ratio and reactivity, often result in unpredictable interactions with, and transformations by, biological and ecological systems [9–10]. Traditional risk assessment approaches, while valuable, are resource intensive and inadequate to fully address the dynamic risks associated with ENMs and their myriad nanoscale forms (i.e., different sizes, geometries, coatings) [11]. The need for more proactive and efficient methodologies has led to the emergence of the Safe and Sustainable by Design (SSbD) framework, which integrates safety considerations throughout the nanomaterial lifecycle, from design to disposal [12–14].

The SSbD concept is closely aligned with the EC Joint Research Centre SSbD framework , the European Chemical Industry Council (Cefic) “Safe and Sustainable by Design” initiative [15–18], the broader agenda of the European Commission on safe and sustainable design for chemicals and advanced materials as part of the EU Green Deal [19] and the EU Chemicals Strategy for Sustainability [20], as well as the work of the OECD Working Party on Manufactured Nanomaterials (WPMN) Steering Group [21].

These frameworks strive to ensure that ENMs and chemicals undergo rigorous evaluation and transparent reporting of hazards, exposures, and life cycle impacts from the earliest stages of product conception. Recent advances in machine learning (ML) and artificial intelligence (AI) have significantly expanded the capabilities of SSbD by enabling high-throughput and automated approaches that can quickly evaluate the safety profile of candidate materials [22] as well as multi-criteria decision analysis in which several parameters (e.g., functionality, safety, sustainability, and cost) are optimised in parallel, thereby accelerating the design of both safe and sustainable nanomaterials [23]. Good data management approaches are of paramount importance to maximise and verify the applicability of novel approaches involving AI and ML.

**Figure 1:**
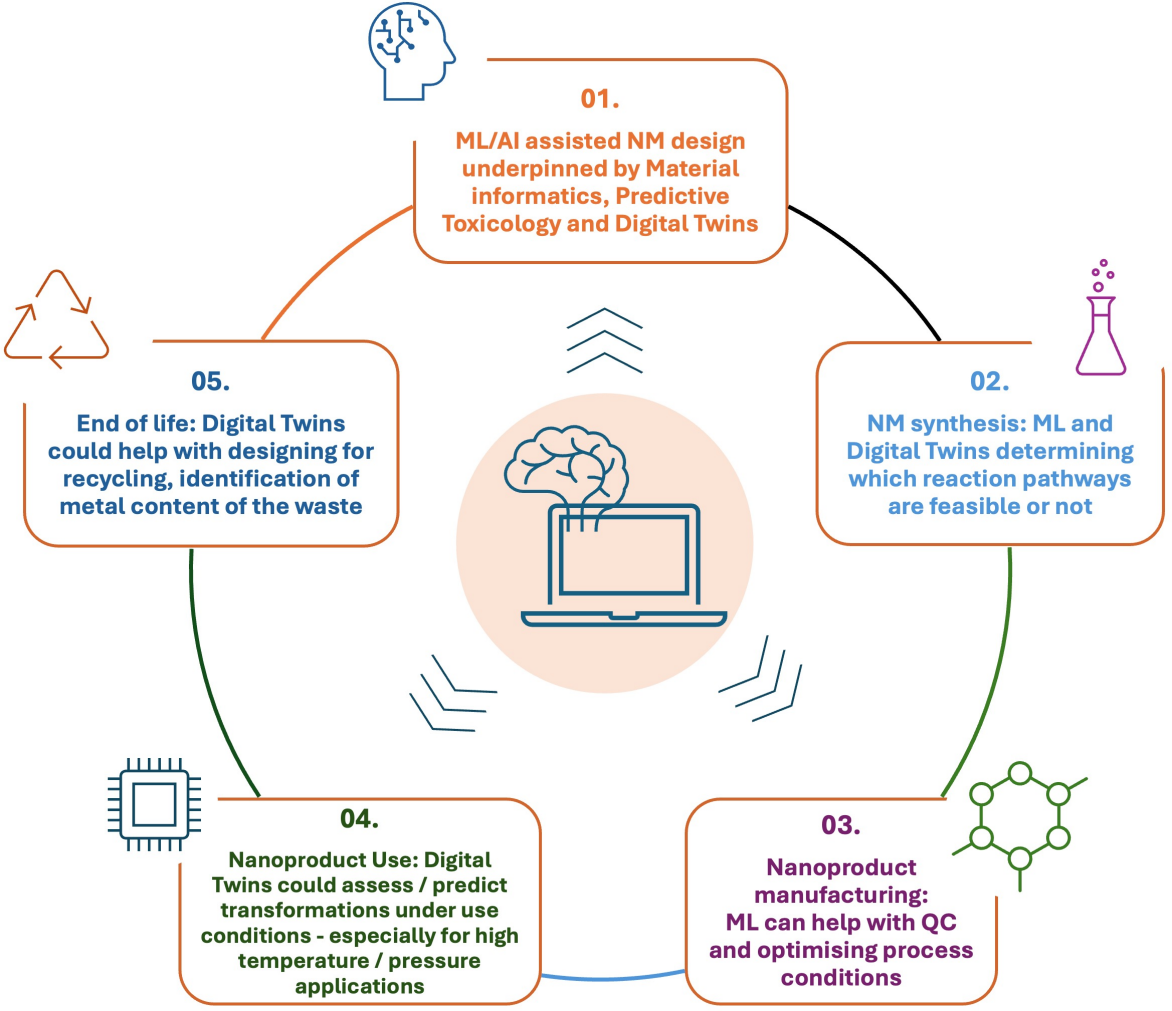
Nanomaterial life cycle underpinned by AI.

On a practical level, ML/AI offers several complementary benefits within SSbD. First, predictive modelling tools, such as quantitative structure–activity relationship (QSAR) models, can forecast toxicological and physicochemical properties of emerging substances, reducing the reliance on time-consuming and costly experimental assays [24–25]. The effectiveness of ML/AI models for nanomaterials is often hindered by inconsistent and non-harmonized physicochemical data. Thus, improving data quality through standardization, metadata annotation, and curated databases is crucial to enhance the reliability and regulatory acceptance of predictions. Second, AI-driven platforms utilizing deep learning techniques enable real-time processing of dynamic sensor data within Internet-of-Things (IoT) environments, facilitating enhanced monitoring and analysis across various applications, including industrial processes [26]. These insights help identify and mitigate potential EHS risks as they evolve, ensuring proactive rather than reactive risk management. Third, dynamic simulations – including digital twin technologies – provide a virtual environment for researchers to run “what if” scenarios, allowing them to explore the impact of variable parameters (e.g., pH, temperature, surface coating) on nanomaterial behaviour in complex biological or ecological systems [27]. Examples of AI implications within the NM life cycle are depicted in Figure 1.

Crucially, these AI-driven methods harmonize with the SSbD frameworks by embedding safety and sustainability considerations within computational workflows, ensuring that industries are better positioned to meet evolving regulatory requirements, fulfil societal expectations for sustainable innovation, and streamline product development cycles [28]. Such integration also paves the way for collaborative, transparent data-sharing networks, where standardized information on nanomaterial properties and toxicity profiles can be used to train increasingly robust ML models. Overall, the synergy between the SSbD concept, advanced ML/AI algorithms, and comprehensive regulatory directives fosters a future-oriented model of nanotechnology development – one that secures both innovation and safety.

## Perspective

### Safe and sustainable by design

Safe and sustainable by design can be defined as “a pre-market approach to chemicals and materials design that focuses on providing a function (or service), while avoiding volumes and chemical and material properties that may be harmful to human health or the environment in particular groups of chemicals likely to be (eco)toxic, persistent, bio-accumulative, or mobile. Overall sustainability should be ensured by minimizing the environmental footprint of chemicals and materials in particular in relation to climate change, resource use, and protecting ecosystems and biodiversity, adopting a lifecycle perspective” (adapted from [12]). Emphasis on early-stage risk assessment contrasts with more reactive approaches [29], which often identify and attempt to address safety issues only after a material or product has already been designed and introduced to the market. By integrating toxicological, ecological, and exposure considerations upfront, SSbD endeavours to minimize hazards while preserving – or even enhancing – functional performance.

In addition to aligning with global regulatory frameworks such as the European Union’s chemical safety regulations and international guidelines for nanomaterials, efforts to operationalize the SSbD framework continue to evolve across research, industry, and regulatory domains. Several key areas require further attention to ensure the effective integration of safety and sustainability considerations into nanomaterial development.

#### Need for harmonized testing protocols

Establishing standardized and reproducible methodologies for characterizing nanomaterial properties – such as size distribution, surface chemistry, and toxicity profiles – is essential. A unified approach to testing under controlled laboratory conditions would enable more reliable cross-comparison of data and enhance confidence among researchers, industry stakeholders, and regulatory bodies [21,30–31].

#### Development of standardized data-sharing frameworks

A major challenge in SSbD implementation is the ability to integrate and share vast amounts of experimental and computational data for diverse ENMs. There is a growing need for interoperable databases and digital platforms that adhere to the FAIR (findable, accessible, interoperable, and reusable) principles, ensuring seamless access to information for researchers and policymakers and ensuring transparency and thereby trust in the assessment outcomes [32–34].

#### Strengthening interdisciplinary collaboration

Greater coordination between academia, industry, and regulatory agencies is needed to comprehensively address environmental, health, and safety concerns. Bringing together toxicologists, materials scientists, engineers, and policymakers would support the alignment of SSbD strategies with evolving legislative requirements, including classification and labelling regulations for chemical substances, including facilitating the development of a common understanding of SSbD with clear definitions, terminology, and criteria [35].

Advancing these areas would contribute to the safe and sustainable development of nanomaterials, ensuring that innovation progresses in a way that meets regulatory expectations and public health priorities.

### Role of ML/AI for scalability and complexity

The increasing complexity of ENMs calls for advanced, data-driven computational tools to enhance analysis and decision-making. ML and AI play a crucial role in this effort, offering powerful capabilities for: (1) Predictive toxicology: AI-driven quantitative structure–activity relationship (QSAR) models can identify potentially hazardous properties of new ENMs before they are synthesized, reducing the need for extensive animal testing and accelerating the design cycle [36–37]. Similarly, AI can support the development of sustainable ENMs through integration of environmental and climate data with information on the production, release, exposure, and toxicity of materials with many complex descriptors [38]. (2) Big data analytics: Advanced algorithms can carefully analyse high-dimensional datasets, identifying patterns between physicochemical characteristics of ENMs, their interactions with biomolecules and toxicity endpoints that may be overlooked by traditional methods [39–41]. (3) Lifecycle modelling: AI-assisted simulations and probabilistic methods support comprehensive lifecycle analyses including prospective approaches, evaluating environmental fate and transport of ENMs, as well as potential occupational and consumer exposures across production, use, and disposal stages [42–44].

### Predictive toxicology

Predictive toxicology is pivotal to SSbD strategies because it enables early-stage assessments of potential nanomaterial hazards, thereby minimizing reliance on time-consuming and ethically challenging animal studies. ML and AI methods form the backbone of these predictive capabilities, allowing researchers to exploit large datasets encompassing everything from physicochemical descriptors to biomolecule interactions to transcriptomic and proteomic information.

QSAR models, for instance, rely on known correlations between specific nanomaterial properties – such as size, shape, and surface chemistry – and various toxicity endpoints. By identifying hazardous materials well before synthesis, QSAR-based screening saves resources, decreases late-stage failures, and aligns with the 3Rs principle (Replacement, Reduction and Refinement), favouring in silico and in vitro approaches over animal testing. The emergence of deep learning techniques, including convolutional neural networks (CNNs) and recurrent neural networks (RNNs), has further heightened the power of predictive toxicology. These advanced algorithms excel in handling high-dimensional data, often integrating transcriptomic and proteomic information to pinpoint molecular pathways responsible for adverse biological outcomes, and linking these molecular changes as a sequence of key events into an adverse outcome pathway [45]. This mechanistic insight, in turn, guides the design of safer nanomaterials by helping researchers engineer specific surface modifications or tailor release profiles to mitigate toxicity. A particularly notable impact of ML/AI models in this arena is their capacity to reduce the extent of in vivo testing while enhancing both the speed and reliability of risk assessments. This capability not only accelerates the innovation cycle but also aligns with regulatory and ethical pressures to identify alternatives to animal experimentation. These tools seamlessly integrate into the SSbD framework, offering proactive detection of potentially hazardous materials or formulations at the earliest stages of research and development. By providing rapid, data-driven feedback on the probable safety profile of a material, predictive toxicology ensures that corrective measures – such as surface functionalization, doping strategies, or substituting alternative compounds – are implemented prior to commercialization. Overall, the synergy between predictive toxicology and SSbD underscores a forward-looking commitment to responsible, sustainable nanotechnology, as these computational methods help deliver materials that meet performance demands without compromising human health or the environment.

### Materials informatics

To date, materials informatics has been predominantly focussed on optimizing functionality, largely through materials acceleration platforms (MAPs) that combine automation, high-throughput experimentation, and ML to accelerate materials discovery [46]. In addition, materials informatics applies advanced data-driven techniques to systematically search the vast chemical and structural design space of engineered nanomaterials, allowing researchers to pinpoint formulations that offer both optimal performance and a reduced risk profile [47–48]. By combining high-throughput computational screening with experimental data, this approach enables rapid candidate selection for diverse applications, from catalysis to targeted drug delivery [49–50]. One of the most powerful aspects of materials informatics lies in its ability to integrate machine learning with multiscale simulation tools – ranging from molecular dynamics to density functional theory – which helps researchers correlate nanoscale features such as particle size, shape, and surface functionalization with macroscopic properties such as catalytic efficiency, biocompatibility, or environmental persistence. This synergy not only speeds up the discovery process but also allows for continuous refinement of computational models as new data emerge from iterative experimental validation. Moreover, inverse design techniques push this paradigm further by autonomously generating candidate compositions that meet predefined targets for both functionality and safety, thereby reducing the trial-and-error components of materials development [51]. In practice, these AI-driven methods can flag potentially hazardous attributes early in the design cycle, enabling prompt adjustments to chemical composition or synthesis protocols that mitigate toxicity without compromising performance. Through such feedback loops, materials informatics cultivates a forward-looking approach to nanomaterial innovation, where safety considerations are integrated at the outset, streamlining the path from virtual screening to commercial deployment.

### Lifecycle analysis

Lifecycle analysis (LCA) offers a holistic framework for assessing environmental, health, and safety implications of engineered nanomaterials at every stage of their existence, beginning with raw material synthesis and continuing through usage, recycling, and eventual disposal. ENMs may undergo transformations such as agglomeration, chemical reactions, or changes in surface properties. These transformations may happen in different environmental and biological contexts, including in air and water under high temperature and pressures and following release and uptake by biota [9]. Therefore, LCA must account for the entire lifecycle of these materials, from production and usage for which industrial materials can often be under extreme conditions (high temperatures, pressures and/or cycling of these) to disposal or recycling, while also capturing the associated uncertainties.

The use of ex-ante and prospective LCA represents a significant advance in sustainability analysis, particularly for emerging technologies such as engineered nanomaterials. Unlike conventional retrospective LCAs, these forward-looking approaches allow researchers and policymakers to anticipate environmental and health impacts before full-scale production or commercialization, enabling more informed design and investment decisions. They are especially relevant in the context of SSbD, where early-stage assessments help minimize environmental burdens and align innovation with long-term sustainability goals. Integrating scenario development, uncertainty analysis, and dynamic system modelling, prospective LCAs support strategic planning and risk mitigation throughout the innovation lifecycle [52].

In parallel, Bayesian models and probabilistic methods have become essential for handling incomplete or fluctuating datasets, allowing analysts to quantify the uncertainty around key factors such as release rates, exposure scenarios, and degradation kinetics [53]. These advanced statistical techniques yield more reliable and transparent LCA outcomes, which in turn enable regulators, industries, and other stakeholders to make informed decisions about the safety and sustainability of nanomaterial applications. Complementing the probabilistic approaches, dynamic modelling tools enable researchers and policymakers to simulate how ENMs behave over time, guiding strategies for safe disposal and recycling [54]. Such tools consider factors such as nanomaterial persistence, potential bioaccumulation in ecosystems, and the efficacy of waste treatment processes, helping to pinpoint when and where SSbD interventions may be most critical. By integrating real-time data on ENM fate and transport, these models provide the flexibility to adapt to new evidence or change regulatory thresholds. Taken together, LCA methodologies – particularly those enhanced by Bayesian and dynamic modelling – support a preventative, SSbD mindset. By illuminating the hidden risks that can arise across the lifespan of a material, they help ensure that nanotechnological innovations do not inadvertently compromise human health or ecological balance.

### Digital twins in safe by design

Digital twins represent a significant leap in SSbD methodologies because they function as high-fidelity, dynamic replicas of physical systems, allowing researchers to explore the behaviour of nanomaterials across a spectrum of virtual scenarios [55]. By pairing experimental inputs (e.g., physicochemical data, toxicity endpoints) with computational models (ranging from physics-based to data-driven models), these digital counterparts evolve in real time as new data and conditions are introduced. This continuous feedback loop not only reduces the need for extensive lab testing, but also accelerates design iterations by highlighting, early on, the potential interactions and risks associated with specific ENMs [56]. One illustrative application involves modelling nanoparticle–protein interactions, a critical factor in drug delivery systems, where digital twins can accurately predict protein adsorption patterns on nanoparticle surfaces through read-across and interpolation from limited experimental datasets [57]. Given that protein corona formation [58] can drastically alter the biodistribution and immunological profile of a nanoparticle, digital twins help pinpoint safer design parameters – such as surface coatings or particle size modifications – which improve biocompatibility. Similarly, in the field of environmental risk assessment, digital twins simulate how ENMs disperse under varying climatic and ecological conditions and advanced environmental fate models can be utilised to explore the impact of changing conditions or application of mitigation or environmental remediation measures on the particle concentrations in specific environmental compartments (e.g., [59] and made accessible via a web application at https://sb4n.cloud.nanosolveit.eu/). These models integrate geospatial data, fluid dynamics, and chemical reactivity, offering a geographically and temporally detailed picture of how ENMs move through – and possibly accumulate in – soil, water, and air. By enabling stakeholders to test “what-if” scenarios, such as accidental spills or long-term usage in consumer products, digital twins enhance predictive accuracy and decision-making regarding waste management, recycling, and potential remediation strategies. Collectively, digital twin technologies embody the core principles of SSbD: prevention, iteration, and information. They provide a living laboratory in silico, where scientists, industry representatives, and policymakers can validate and refine nanomaterial safety profiles long before real-world deployment, fostering a more responsible and sustainable innovation landscape. A number of web applications for construction of digital nanomaterials have also been made available recently to support the implementation of digital twins and enable users with limited programming or informatics skills to apply these technologies, including NanoConstruct [36], ASCOT [60], and NanoTubeConstruct [61]. Beyond material design, digital twins can also be applied to simulate and predict occupational exposure scenarios, helping ensure that manufacturing processes are not only efficient but also protective of worker health and safety. This makes them a valuable asset across the full SSbD framework, addressing both environmental and human health dimensions [62].

### Challenges and opportunities

The integration of ML/AI and digital twin technologies within SSbD paradigms presents both significant challenges and opportunities, particularly as the field moves from conceptual demonstrations to large-scale industrial implementation and regulatory adoption. One of the most pressing issues is the availability and quality of data, as many current nanomaterial datasets are fragmented, inconsistently formatted, and insufficiently annotated for robust ML/AI model training [63–64]. Moreover, these datasets often arise from disparate sources – academic research labs, industrial R&D facilities, and public databases – each with its own protocols and measurement standards. Such heterogeneity complicates efforts to systematically integrate and compare results, thereby limiting the accuracy and generalizability of predictive models. Addressing this challenge necessitates concerted efforts to create FAIR-compliant nanoinformatics databases [63]. By adopting standardized metadata schemas, controlled vocabularies, and transparent data-sharing agreements, stakeholders can facilitate more seamless collaboration and unlock the full potential of AI-driven risk assessment. Progress is being made in this direction through application of big data curation and development of modelling friendly nanostructure annotations [65] and modelling-ready nanomaterials EHS and SSbD relevant databases including VINAS [66] and NanoPharos [67].

**Figure 2:**
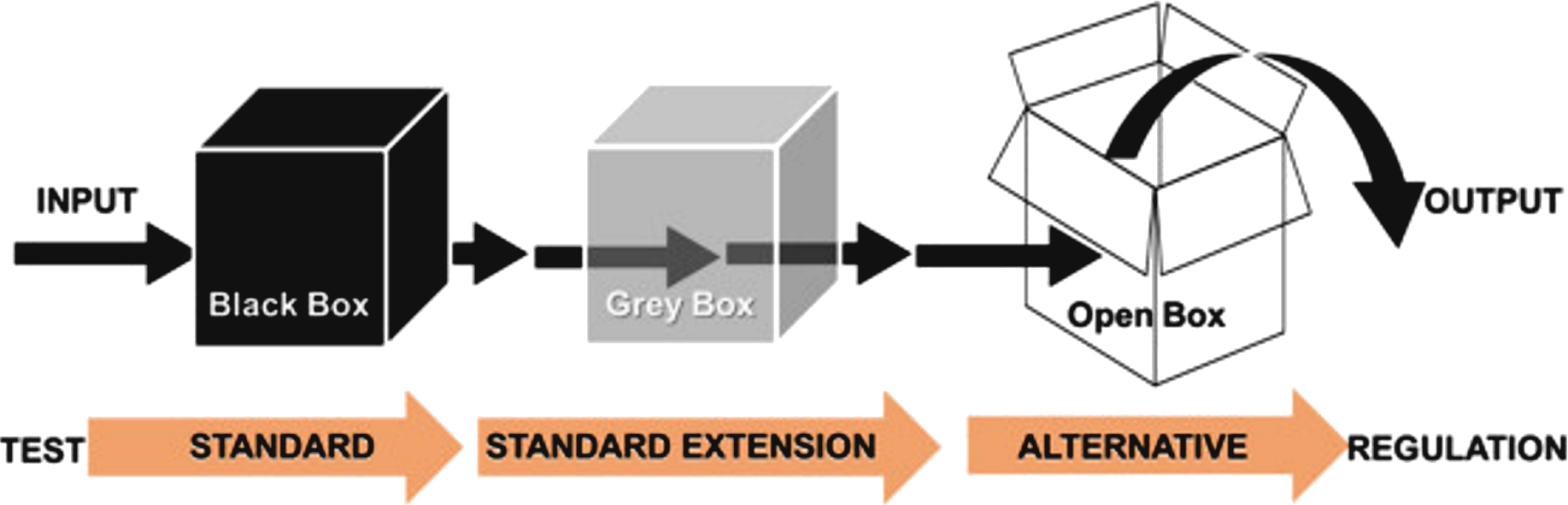
A schematic representing how adding new endpoints or using alternative (non-animal) test methods, including in silico approaches, can help reveal the underlying mode of action. These additional methods make it possible to “open the black box” of traditional apical endpoints, which only show the effects but not their causes. Figure 2 reproduced from [69] (© 2021 S. I. L. Gomes et al., published by Elsevier Ltd., distributed under the terms of the Creative Commons Attribution-NonCommercial-NoDerivatives 4.0 International Public License, https://creativecommons.org/licenses/by-nc-nd/4.0/). This content is not subject to CC BY 4.0.".

Another major hurdle is model interpretability, particularly for deep learning approaches that often function as “black boxes”. Despite their high predictive power, complex architectures such as convolutional neural networks or recurrent neural networks can obscure how a model reaches specific toxicity or exposure predictions. This lack of transparency can undermine regulatory trust and slow adoption in safety-critical domains, as stakeholders – including policymakers, industry representatives, and the broader public – require a clear understanding of the origin and quality of (in silico) results and how decisions are made. The emerging field of explainable AI (XAI) offers promising solutions by developing methods (e.g., SHAP values, LIME, and gradient-based techniques) that highlight which input variables most strongly influence the output of a model. Adopting XAI frameworks also presents an opportunity to refine model architectures by ensuring they align more closely with known mechanistic or toxicological pathways, thereby bridging the gap between computational insights and domain expertise. Despite these obstacles, the future holds considerable opportunities. As the volume of high-quality, standardized data grows, ML algorithms will become more capable of identifying complex structure–property–toxicity relationships, potentially accelerating the safe commercialization of next-generation nanomaterials [68]. Similarly, advances in XAI approaches will strengthen regulatory acceptance by providing transparent, well-justified predictions that can be validated against experimental data or well-established mechanistic models. It has been suggested that the current regulatory approach, relying on animal tests that measure outcomes such as mortality without explaining the underlying mechanisms, is effectively a “black box.” In contrast, using AI and XAI can provide mechanistic insights, leading to greater transparency for regulators and improved protection for the public [69]. Increasing the standardisation of approaches for documenting models is essential for regulatory acceptance. Towards this goal, the Easy-MODA tool [70] used to describe ML/AI models, serves a similar purpose to the QSAR Model Reporting Forms used for QSAR models. At the same time, ongoing progress in digital twin technologies – particularly those incorporating real-time sensor data – enables adaptive feedback mechanisms that support proactive decision making. This comprehensive integration of data standards, explainable AI, and digital twins has the potential to not only optimize product development cycles but also to enhance public confidence, fostering an innovation ecosystem where safety and sustainability are fundamental to technological progress. While ML models are often referred to as being a black box, a recent paper up-ended this conception, suggesting that the current gold-standard of in vivo apical end-point tests are the black box (see Figure 2). They provide no mechanistic insights to explain the observed impacts. However, extending traditional animal tests with approaches such as toxicogenomics analyses increases the transparency of the box (system). Incorporating alternative test methods (also called new-approach methodologies or NAMs), and which include in silico (computational) assessment, can fully “open the box”, revealing mechanistic drivers and enabling establishment of dose-response relationships, read-across, and other insights that allows regulators to gain a deeper understanding in comparison to what is possible with the standard approach alone.

The future of SSbD in nanotechnology will likely be driven by hybrid modelling frameworks that unite ML/AI techniques with physics-based simulations, creating a more precise and scalable approach to nanomaterial risk assessment [71]. By coupling data-driven algorithms – capable of rapidly processing high-dimensional, heterogeneous datasets – with the fundamental insights provided by mechanistic and thermodynamic models, these hybrid systems will enable researchers to predict both performance and toxicity under a broader range of conditions. This exchange of knowledge between computational paradigms not only improves predictive accuracy but also enhances generalizability, as models can be continuously updated with new empirical data. In parallel, the development of interconnected digital twin ecosystems has the potential to significantly streamline SSbD workflows, from initial design concepts all the way to industrial-scale manufacturing [1]. Rather than working in isolated environments, researchers, engineers, and quality-control teams will be able to share real-time, sensor-driven data within dynamic virtual platforms, allowing for rapid adjustments to nanomaterial formulations or processing parameters in response to emerging safety or efficacy concerns. By simulating how nanomaterials behave across varying operational scenarios – incorporating factors like temperature, pH, or mechanical stress – digital twins will facilitate safer and more efficient scaling of novel ENMs. Achieving these goals – namely, safer nanomaterial design, more efficient SSbD workflows, and scalable implementation – requires well-defined policy frameworks that incorporate AI-derived insights to ensure transparency, foster regulatory trust, and align technological innovation with public health and environmental protection.

Policymakers must work closely with industry and academic partners to implement adaptive regulations. Collaborative initiatives – in which stakeholders openly share data, best practices, and methodologies – will be essential to fostering a transparent, socially responsible nanotechnology landscape. Through the convergence of hybrid modelling, digital twins, and informed policy, SSbD can continue to evolve into a powerful catalyst for safer, more sustainable innovation in the nanoscale area.

## Conclusion

ML and AI, in concert with digital twin technologies, are fundamentally reshaping the SSbD paradigm by elevating the speed, depth, and precision of nanomaterial risk assessment. Through predictive toxicology, these computational tools can rapidly forecast hazardous characteristics of newly conceived materials, reducing both resource expenditures and ethical concerns associated with animal testing. Materials informatics extends this impact by applying ML to analyse large chemical and structural datasets, enabling the efficient discovery of nanomaterials that achieve an optimal balance between high performance, green synthesis routes, and minimized toxicity. Moreover, digital twins contribute a real-time, iterative layer of validation and optimization, enabling researchers to virtually explore a variety of scenarios – from nanoparticle–protein interactions to environmental dispersion without ever having to synthesize the candidate materials until the final optimised one – while continuously refining design parameters in response to new data. However, this technologically advanced ecosystem still faces some critical hurdles to implementation. One major challenge is the complex and interdisciplinary nature of nanotechnology, which demands not only advanced computational models but also a deep mechanistic understanding of nano–bio interactions, environmental fate, and lifecycle behaviour – areas where current models often fall short. Additionally, implementation of the SSbD framework requires a holistic integration and optimization of functionality, safety, and sustainability across the entire life cycle of a material, from design and production to use and disposal. Realizing this vision requires more than FAIR data principles alone; it necessitates harmonized data sheets for key toxicological and ecotoxicological endpoints, standardized test methods, and physicochemical characterization protocols, and the development of nano-specific life cycle inventory data suitable for reliable LCAs. Without these foundational elements, even the most sophisticated ML models may yield biased or non-transferable results. Efforts to develop FAIR-compliant data infrastructures and interpretable ML models will thus be critical to accelerating the adoption of the SSbD principles at industrial and policy levels. Interdisciplinary collaboration among academia, government agencies, and private industry can turn computational advances into real-world solutions that protect both people and the environment. The future of safer, sustainable nanotechnology depends on this collaboration – using predictive tools, digital twins, and smart regulations to create high-performing materials that are produced in ethical and responsible ways.

## Data Availability

Data sharing is not applicable as no new data was generated or analyzed in this study.
